# Design of N‐Type Textured Bi_2_Te_3_ with Robust Mechanical Properties for Thermoelectric Micro‐Refrigeration Application

**DOI:** 10.1002/advs.202206395

**Published:** 2022-12-29

**Authors:** Yu‐Ke Zhu, Yifan Jin, Jianbo Zhu, Xingyan Dong, Ming Liu, Yuxin Sun, Muchun Guo, Fushan Li, Fengkai Guo, Qian Zhang, Zihang Liu, Wei Cai, Jiehe Sui

**Affiliations:** ^1^ State Key Laboratory of Advanced Welding and Joining Harbin Institute of Technology Harbin 150001 P. R. China; ^2^ School of Materials Science and Engineering Institute of Materials Genome & Big Data Harbin Institute of Technology Shenzhen 518055 P. R. China

**Keywords:** bismuth telluride, machinability, micro‐refrigeration, preferred orientation, quality factor

## Abstract

Thermoelectric refrigeration is one of the mature techniques used for cooling applications, with the great advantage of miniaturization over traditional compression refrigeration. Due to the anisotropic thermoelectric properties of n‐type bismuth telluride (Bi_2_Te_3_) alloys, these two common methods, including the liquid phase hot deformation (LPHD) and traditional hot forging (HF) methods, are of considerable importance for texture engineering to enhance performance. However, their effects on thermoelectric and mechanical properties are still controversial and not clear yet. Moreover, there has been little documentation of mechanical properties related to micro‐refrigeration applications. In this work, the above‐mentioned methods are separately employed to control the macroscopic grain orientation for bulk n‐type Bi_2_Te_3_ samples. The HF method enabled the stabilization of both composition and carrier concentration, therefore yielding a higher quality factor to compare with that of LPHD samples, supported by DFT calculations. In addition to superior thermoelectric performance, the HF sample also exhibited robust mechanical properties due to the presence of nano‐scale distortion and dense dislocation, which is the prerequisite for realizing ultra‐precision machining. This work helps to pave the way for the utilization of n‐type Bi_2_Te_3_ for commercial micro‐refrigeration applications.

## Graphical abstract

Commonly used methods for increasing the preferred orientation of n‐type Bi_2_Te_3_ include liquid phase hot deformation (LPHD) and traditional hot forging (HF). This work, as an insightful understanding of the HF method, discloses the intrinsic mechanisms of both superior thermoelectric and robust mechanical properties, aiming to shed light on texture engineering utilized for viable micro‐refrigeration applications.

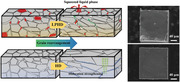

## 1 Introduction

The Peltier effect, which was discovered in the mid of 18^th^ century, renders thermoelectric refrigeration as the alternating cooling path for traditional compressor refrigeration.^[^ ^1^ ^]^ In real practical applications, its main characteristics of quick response, precise temperature control, as well as small size and lightweight become the absolute predominance to compete with other refrigeration technologies.^[^ ^2^ ^]^ Specifically, the micro‐refrigeration application using thermoelectric modules has unprecedentedly attracted both fundamental and practical interest.^[^ ^3^ ^]^

However, the relatively low conversion efficiency and poor mechanical properties still restrain the large‐scale commercialization of thermoelectric micro‐refrigeration.^[^ ^4^ ^]^ Therefore, developing advanced thermoelectric materials is of significance to simultaneously realize good thermoelectric and robust mechanical properties.^[^ ^5^ ^]^ The dimensionless thermoelectric merit *ZT*, defined as *ZT* = *α^2^σT/κ*, enables the evaluation of thermoelectric properties for materials,^[^ ^6^ ^]^ while the mechanical properties can be accessed by machinability, hardness, bending, and compressive strength.^[^ ^5^, ^6^, ^7^ ^]^ Typical high‐performance thermoelectric materials, such as Bi_2_Te_3_,^[^ ^8^ ^]^ GeTe,^[^ ^6^, ^9^ ^]^ PbTe/Se,^[^ ^4^, ^6^, ^10^ ^]^ Cu_2_S/Se^[^ ^11^ ^]^ and half Heusler,^[^ ^12^ ^]^ show *ZT* peaks at different temperature ranges that is mainly caused by the different band gap. Bi_2_Te_3_ system is the dominant material in thermoelectric refrigeration applications due to its best thermoelectric properties at room temperature. Unfortunately, the polycrystalline n‐type BiTeSe alloys show inferior thermoelectric properties compared to p‐type BiSbTe.^[^ ^13^ ^]^ Regarding making modules and systems, single crystal n‐type BiTeSe, despite the good thermoelectric performance, can not suffer the subsequent machining, processing, and fabrication process because of the cleavage plane along the van der Waals layer.^[^ ^14^ ^]^

**Figure 1:**
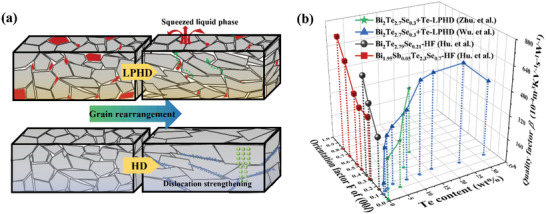
a) The schematic images of the sintering process for LPHD and HF. b) The relationship among quality factor *β*, Te content, and preferred orientation factor *F* along (00*l*) direction, where the data are collected from the representative works in texture engineering for n‐type Bi_2_Te_3_ alloys.^[28‐31]^

Due to the layered feature of crystal structure, texture engineering can effectively construct specific orientations to enhance thermoelectric properties for n‐type Bi_2_Te_3‐_ *_x_* Se*_x_* alloys.^[^ ^15^ ^]^ Among these reported texturing methods, hot forging (HF) and liquid phase hot deformation (LPHD) are the most commonly used strategies,^[^ ^16^ ^]^ which lead to the increased carrier mobility and *ZT* value due to the increment of orientation along (00*l*) direction (**Figure** **1**a). HF method was performed for the zone‐melting and hot‐pressing sample of Bi_1.95_Sb_0.05_Te_2.3_Se_0.7_, leading to a high *ZT* of 1.3.^[^ ^17^ ^]^ Regarding the LPHD method, Te‐rich eutectic phases are melted as liquid phase and squeezed out of graphite die, which also enables the grains rearrangement towards the perpendicular direction to pressure and therefore realizes the enhancement of intrinsic carrier mobility.^[^ ^18^ ^]^ Zhu et al. adopted the LPHD method together with Se/Sb alloying and achieved a record *ZT* of 1.4.^[^ ^19^ ^]^ The original data of their samples was used to calculate the quality factor *β* defined as *β = µ*_0_(*m* ^*^/*m*_e_)^3/2^/*κ*_L_, which largely removes the effect of doping level to access the inherent thermoelectric potential of materials.^[^ ^20^ ^]^ Here, *µ*_0_ is the intrinsic carrier mobility, *m* ^*^ is the density of state effective mass and *κ*_L_ is the lattice thermal conductivity.^[^ ^21^ ^]^ In the three‐dimensional coordinates system consisting of Te content, orientation factor *F* along (00*l*), and *β*, it was found that *F* has a positive linear correlation with *β* (Figure 1b). However, it is challenging to elucidate which method is better to increase *β*. It also lacks a comprehensive comparison of mechanical properties and machinability between these two methods for micro‐refrigeration applications, especially for the underlying mechanisms.^[^ ^16^, ^22^ ^]^

In this work, both LPHD and HF methods resulted in the preferred grain orientation of Bi_2_Te_2.9_S_0.1_ bulk sample, but they played a distinctive role in microstructure evolution and thermoelectric properties. We performed the parallel comparison between the above two methods to identify the full benefits of hot forging in both thermoelectric and mechanical properties for micro‐refrigeration applications. For LPHD samples, the Te‐rich chemical potential reduced the density of state effective mass and simultaneously increases the carrier concentration, revealed by the defect formation energy calculations, leading to the counterbalanced enhancement of *ZT*. In contrast, hot forging method not only realizes the stronger preferred orientation with an optimum carrier concentration, but also suppresses the lattice thermal conductivity by high‐density dislocation scattering. In addition to the good thermoelectric performance, HF samples also exhibited robust mechanical properties and good micro‐machinability, which shed light on the feasible micro‐refrigeration applications using n‐type Bi_2_Te_3_‐based materials.

## 2 Experimental Section

### 2.1 Synthesis

High purity raw materials Bi (bulk, 99.99%, Alfa Aesar), Te (bulk, 99.99%, Alfa Aesar), S (bulk, 99.99%, Alfa Aesar) were weighed according to the nominal composition of Bi_2_Te_2.9+_ *_x_* S_0.1_ (*x* = 0, 0.015, 0.03, 0.06 and 0.12) in the glove box, and were loaded into quartz tubes sealed under a vacuum condition of 10^−3^ Pa. The sealed quartz tubes were heated to 1073 K maintaining for 10 h with a natural cooling process. After melting, the ingot was crushed to powders by high‐energy ball milling method (SPEX 8000 M, America) with a high purity nitrogen atmosphere, under 875 rpm min^−1^, 2 h. For LPHD samples, their fine powders were loaded into a graphite die with a diameter of 12.7 mm and sintered by spark plasma sintering (SPS) method under 673 K, 80 MPa for 5 min. For HF samples, the as‐sintered bulk was loaded into a graphite die with a diameter of 15 mm for HF1 and 20 mm for HF2, and forged under 733 K, 50 MPa for 5 min. All samples were cut and polished toward the required conditions and all thermoelectric properties were measured along the direction that is perpendicular to SPS pressure.

### 2.2 Characterization

The phase identification was conducted by the X‐ray diffraction (XRD, EmpyreanX, Netherlands) with Cu K*α* (*λ* = 1.5406 Å) radiation. The scanning electron microscopy (SEM, HELIOS Nanolab 600i, America/ SEM, Zeiss Merlin Compact, Germany) and energy dispersive X‐ray spectroscopy (EDS) engaged in the characteristic for micromorphology and identification of elements distribution. The ZEM‐3 (Ulvac‐Riko, Inc. Japan) was adopted to test the electrical conductivity and Seebeck coefficient. The thermal conductivity was obtained by calculation of *κ = DρC_p_*, where *D* is the thermal diffusivity measured by the laser flash method (LFA‐457, Netzsch, Germany), *ρ* is the density measured by the Archimedes method and *C_p_* is the heat capacity obtained through Dulong‐Petit law. The carrier concentration (*n*_H_) and mobility (µ_H_) were calculated by the formula *n*_H_ = 1/(*e* · *R*_H_), and µ_H_ = *σ* · *R*_H_, where *R*_H_ is the Hall coefficient acquired by the van der Pauw technique under a 1.5 T magnetic field. The preferred orientation distribution, grain size, and G.N.D. distribution were performed by electron back‐scattered diffraction (EBSD, Oxford Nordlysnano, England) and analyzed by the software namely ATEX.^[^ ^23^ ^]^ The transmission electron microscopy (TEM, FEI Talos F200X, America) was used to characterize microstructure. The Vickers hardness of all samples was tested by a microhardness tester (HVS‐1000TM/LCD) with a load of 0.5 N for 10 s. The electronic universal testing machine was adopted to check the compressive strength of all samples with the same size of 2 × 2 × 4 mm^3^ under a compressive speed of 0.05 mm min^−1^.

### 2.3 Calculation

The first‐principles calculations are applied to study the defects formation energy (Δ*H*). The projector augmented wave (PAW) pseudo‐potentials were adopted to contact the total energy density functional theory calculations that were implemented in the Vienna ab initio simulation package (VASP) code. A 135‐atom supercell with three 3 × 3 quintuple layers was constructed and used to simulate the status of predominant defects under the Te‐rich condition. The Δ*H* was calculated based on the formula (1):

(1) $$\Delta H=E\left(D^{q}\right)-E_{\mathrm{bulk}}+\sum_{i}\Delta n_{i}\mu_{i}+q\left(E_{F}+E_{VBM}\right)$$

where *E*(*D* ^q^) is the energy of the supercell including the defect in charge state *q, E*_bulk_ is the energy of the initial bulk supercell, and Δ*n*_i_ and Δε_i_ are the change in the number of atoms and chemical potential of element *i*, respectively.

## 3 Results and Discussions

**Figure 2:**
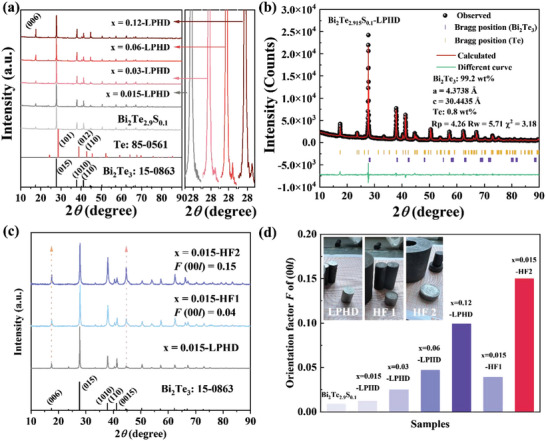
a) XRD patterns of bulk Bi_2_Te_2.9+_ *_x_* S_0.1_‐LPHD (*x* = 0, 0.015, 0.03, 0.06, and 0.12) samples, and b) the Rietveld refinement results of *x* = 0.015‐LPHD sample based on double phases. c) XRD pattern of bulk x = 0.015‐LPHD, *x* = 0.015‐HF1 and *x* = 0.015‐HF2 sample. d) The calculated orientation factor *F* towards (00*l*) direction of all prepared samples.

The X‐ray diffraction patterns of Bi_2_Te_2.9+_ *_x_* S_0.1_‐LPHD (*x* = 0, 0.015, 0.03, 0.06, and 0.12) bulk samples indicate that the major phases are indexed of Bi_2_Te_3_ (15‐0863) with R‐3m space group (**Figure** **2**a). With the increased Te content, a gradually intensified impurity peak corresponding to (101) of Te second phase (85‐0561) is located on the right of (015) main peak of Bi_2_Te_3_. Based on the double‐phase Rietveld refinement, the mass ratio of Te second phase reaches 0.8 wt.% for *x* = 0.015 samples (Figure 2b). It should be mentioned that some materials squeezed out of the graphite die after SPS sintering are observed in *x* = 0.12‐LPHD sample which corresponds to the increased orientation of (006) crystal surface. Therefore, for LPHD samples, it indicates that the prerequisite of the obviously increased orientation of (00*l*) is the occurrence of grain rearrangement induced by a substance squeezed out. Due to the suitable carrier concentration, negligible composition deviation, and the highest *ZT* value, the sample *x* = 0.015‐LPHD has been employed for the further hot‐forging process. The major phase of XRD patterns for *x* = 0.015‐HF samples also are indexed of Bi_2_Te_3_, in which the intensity of both (006) and (0015) crystal surface was stronger. The orientation factor *F* towards (00*l*) direction of all prepared samples was calculated by XRD data and formulas S1‐S3, Supporting Information, shown in Figure 2d.^[^ ^24^ ^]^ Here as expected, both LPHD and HF method can directly induce the alignment of grains and lead to the increased preferred orientation.

SEM images of the fresh fracture morphology indicate an increase in matrix grain size from 0.5 to 5 µm with the increased Te content (Figure S1a‐e, Supporting Information). Corresponding to the XRD results, extra Te content is the unique variable motivating the introduction of Te second phase and Te‐Bi_2_Te_3_ eutectic phase, which has a lower melting point than that of matrix to facilitate the grain growth in the SPS sintering process. The backscattered electron image (BSE) of *x* = 0.015‐LPHD sample presents a homogeneous phase while *x* = 0.12‐LPHD sample presents obvious phase contrast (Figure S1 and S3f,g). The spatial distribution of black areas among the BSE images, SE images, and EDS results indicates that the phase contrast stems from the appearance of eutectic phases and porous structure (Figure S1g‐i, Supporting Information). Due to the hot forging temperature of 733 K which is higher than the melting point of the eutectic phase, the evaporation might be the predominant reason for the porous structure.^[^ ^25^ ^]^

**Figure 3:**
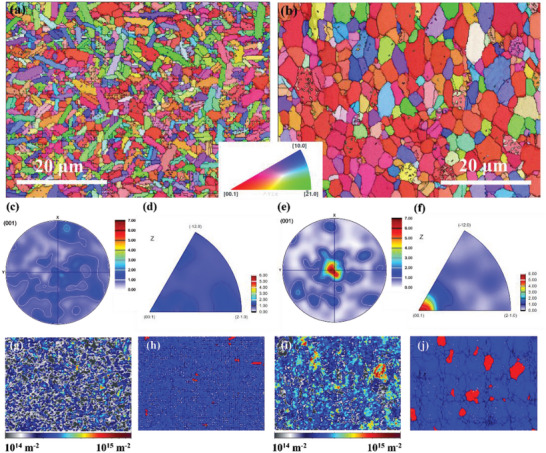
The a,b) Z‐Euler images, c,e) pole figures along (001) direction, d,f) inverse pole figures (IPF), g,i) geometrically necessary dislocations (GND) maps and h, j) grain distribution maps after recrystallization (blue for recrystallized grain, red for sub‐grain) of *x* = 0.12‐LPHD and *x* = 0.015‐HF2 samples, respectively

Furthermore, electron backscattering diffraction (EBSD) has been utilized to analyze the organization, structure, and grain orientation distribution of two typical samples, namely *x* = 0.12‐LPHD and *x* = 0.015‐HF2. The Z‐Euler image of *x* = 0.12‐LPHD sample presents locally distributed bar‐shaped grains which also has been substantially found in the Bi_2_Te_2.7+_ *_x_* Se_0.3_ sample (**Figure** **3**a).^[^ ^18^ ^]^

The bar‐shaped grains are surrounded by lots of equiaxed grains with a smaller size. Due to the migration of the low melting point eutectic phase in the grain boundaries, the initial equiaxed grains would be rotated and stretched along the eutectic phase migration direction. The eutectic phase can also release the stored energy in fine grains produced by high‐energy ball milling to promote grain growth. Therefore, the rearrangement of grains for LPHD samples is seriously dependent on the extra Te content. The Z‐Euler image of 0.015‐HF2 sample shows uniformed equiaxed grains with a larger size than that of *x* = 0.12‐LPHD sample (Figure 3b). There is no obvious bar‐shaped grain observed on the map. The high sintering temperature and largely stored deformation energy are the main factors to motivate grain growth. The results of both pole figures and inverse pole figures present a higher concentrated distribution along the (00*l*) direction in 0.015‐HF2 sample towards the *z*‐axis, that is the SPS pressure direction (Figure 3c‐f). The higher pole density corresponds to the higher orientation factor of XRD results. Furthermore, the geometrically necessary dislocation (GND) map of *x* = 0.015‐HF2 sample possesses stronger intensity than that of *x* = 0.12‐LPHD sample (Figure 3g‐i). As the counterpart of GND map, grain distribution maps after recrystallization indicate that the recrystallization fraction of *x* = 0.12‐LPHD sample is 99.17% and of *x* = 0.015‐HF2 sample is 97.01%. Some sub‐grains can be observed in the *x* = 0.015‐HF2 sample. The higher sintering temperature and larger deformation degree concurrently promoted grain growth and dynamic recrystallization. Therefore, the *x* = 0.015‐HF2 sample simultaneously has a larger grain size and stronger GND.

**Figure 4:**
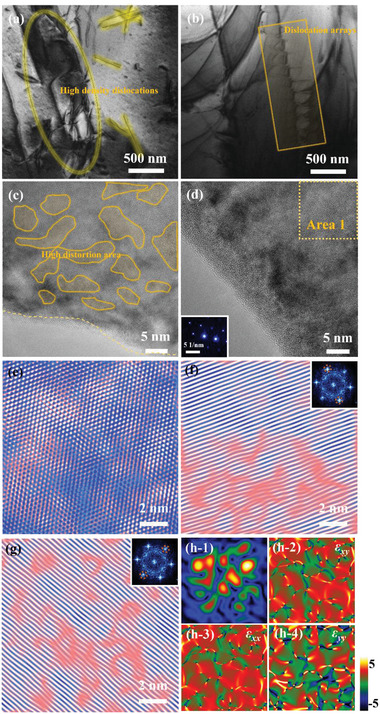
TEM results of *x* = 0.015‐HF2 sample: The a,b) mediate‐magnification TEM images, the c,d) high‐resolution TEM images, e‐g) the inverse Fourier transform (IFFT) image of Area 1 with the inset as fast Fourier transform (FFT). h1‐4) The stress and strain distribution are analyzed by geometric phase images (GPA).

The mediate‐magnification TEM image of *x* = 0.015‐HF2 sample indicates substantial dislocation entanglements (**Figure** **4**a). Dislocation arrays have been found existing in local grain boundaries which is the typical characteristic for the hot forging sample (Figure 4b). In high‐resolution TEM images (HRTEM), it is effortless to observe quantities of high‐distortion areas (Figure 4c). The area #1 along [001] zone‐axis direction in the HRTEM image has been contacted with fast Furious transform (FFT) and inverse Furious transform (IFFT) (Figure 4d). The substantially nano lattice distortions and dislocations can be also found in the IFFT images within different crystal surfaces (Figure 4e‐g). The geometric phase images (GPA) present the three‐dimension distribution of strain field which could potentially render the scattering of phonons and the strengthening of mechanical properties^[^ ^8^, ^26^ ^]^(Figure 4h).

**Figure 5:**
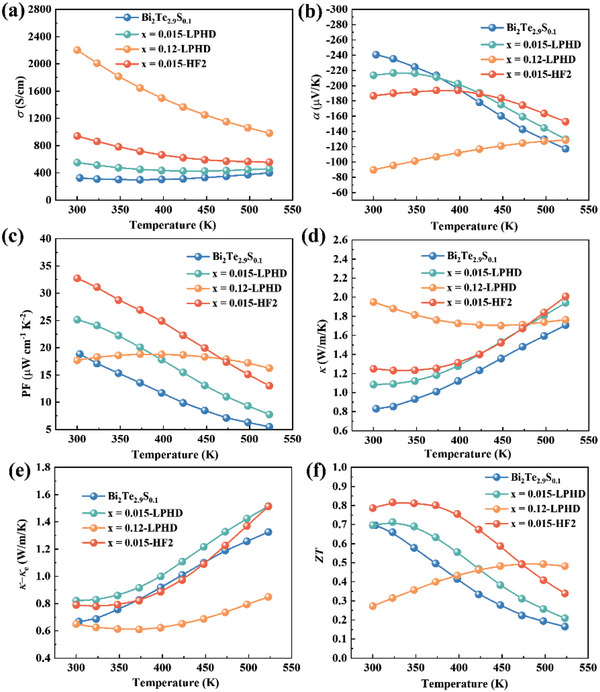
Temperature dependence of thermoelectric properties for samples of Bi_2_Te_2.9_S_0.1_, *x* = 0.015‐LPHD, *x* = 0.12‐LPHD, and *x* = 0.012‐HF2. a) electrical conductivity *σ*, b) Seebeck coefficient *α*, c) power factor *PF*, d) total thermal conductivity *κ*, e) thermal conductivity of *κ* – *κ*_e,_ and f) figure of merit *ZT*, respectively.

Thermoelectric properties of LPHD and HF samples are respectively shown in Figures S2 and S3, Supporting Information. All samples represent a typical behavior of degenerate semiconductors with the temperature dependence of decreased electrical conductivity except the pristine Bi_2_Te_2.9_S_0.1_. The raised Te content and increased HF times both elevate the electrical conductivity and decrease the Seebeck coefficient at room temperature (**Figure** **5**a,b).

The HF method obviously enhances the power factor, while the LPHD method unexpectedly leads to a decrease. The highest power factor in this work reaches 32.5 µW cm^−1^ K^−2^ at 300 K for *x* = 0.015‐HF2 sample (Figure 5c). Likewise, the total thermal conductivity also increases with the raised Te content or the increased HF times for all samples, which is mainly contributed by the elevated electron thermal conductivity and preferred orientation, respectively (Figure 5d). The thermal conductivity *κ*‐*κ*_e_ is roughly equivalent to the lattice thermal conductivity (Figure 5e). Compared to *x* = 0.015‐LPHD sample, the *x* = 0.015‐HF2 sample has a lower *κ*‐*κ*_e_ but with a higher *F* value, which might hinge on the phonon scattering by high dense of dislocations. As expected, the sample of *x* = 0.015‐HF2 in this work possessing the highest orientation factor reaches the maximum *ZT* value of 0.81 at 323 K (Figure 5f).

**Figure 6:**
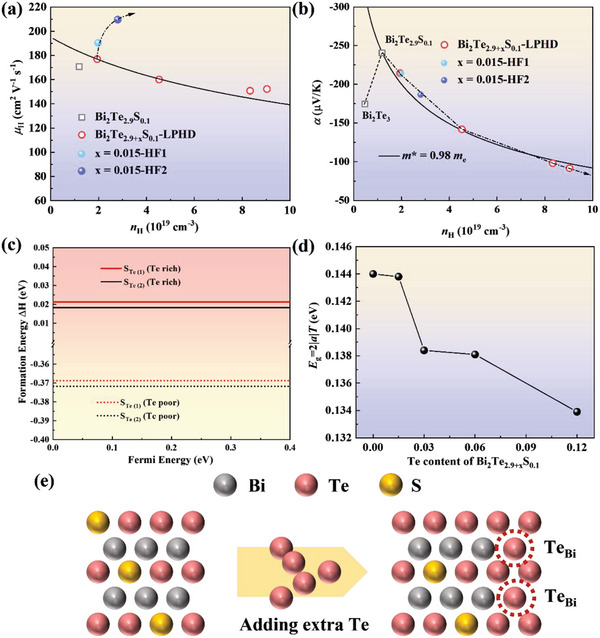
The carrier concentration dependence of a) Hall mobility *µ*_H_ and b) Seebeck coefficient *α*, c) the calculated defects formation energy ΔH of S_Te(1)/(2)_ under Te‐poor and Te‐rich conditions, d) the roughly evaluated band gap, e) the schematic diagram of the decreased S_Te_ concentration.

In order to eliminate the effect of the inherent properties optimization which might be covered by the fluctuated carrier concentration, here Hall measurement is adopted to complement pivotal arguments. The *n*_H_ of LPHD samples increases tremendously when adding excess Te content, so the antisite defects Te_Bi_ could be the only reason. Based on the restructured single parabolic band model, the simulation result of the relationship between *n*_H_ and *µ*_H_ according to the inherent parameters of *x* = 0.015‐LPHD sample is given as the black solid line^[^ ^20b^ ^]^ (**Figure** **6**a). The practical *µ*_H_ of *x* = 0.06/0.12‐LPHD samples deviates from the simulation result that mainly depends on the increased *F*. Due to the larger *F* for HF samples than that of LPHD samples, a much bigger increment of *µ*_H_ is consequently obtained. The *n*_H_ dependence of *α* indicates a reduced density of state effective mass m^*^ from 1.09 *m*_e_ for *x* = 0.015‐LPHD to 0.91 *m*_e_ for *x* = 0.12‐LPHD (Figure 6b). Here, the calculated defect formation energy proves that the formation energy of S_Te_ point defect under Te‐rich chemical potential is obviously higher than that of under Te‐poor condition (Figure 6c). It provides direct evidence that the Te‐rich chemical potential can lead to the lower concentration of S alloying at the anion lattice site. The band gap could be roughly evaluated by *E*_g_ = 2|*α*|*T*. Here, the estimated *E*_g_ for LPHD samples continuously decreases by adding extra Te that is in contrast to the effect of S alloying in Bi_2_Te_3_ (Figure 6d). Normally, a reduced band gap is related to the decreased effective mass and the increased intrinsic carrier mobility. The defect formation energy and decreased band gap offered theoretical and experimental evidence, respectively. Therefore, it can be ascertained that the reduced *m* ^*^ in LPHD samples affects *α*, due to the composition deviation (Figure 6e), while HF samples could maintain the optimized composition a with a higher *m* ^*^ and effectively increase the orientation factor.

**Figure 7:**
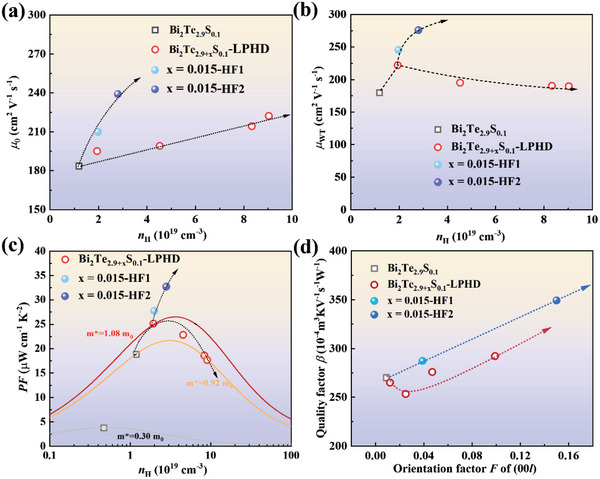
The carrier concentration dependence of a) intrinsic mobility *µ*_0_, b) weighted mobility *µ*_wt,_ and c) power factor *PF*, d) the relation between orientation factor F of (00*l*) and calculated quality factor *β* of LPHD and HF samples.

Mainly due to the increased *F*, the intrinsic mobility *µ*_0_ in both LPHD and HF samples exhibits an obvious increment (**Figure** **7**a). The unchanged *m* ^*^ and increased *µ*_0_ for HF samples synergistically yield much higher weighted mobility, therefore generating an optimistic enhancement on *PF* (Figure 7b,c). However, for LPHD samples, the increased *µ*_0_ can not offset the decrement of *m* ^*^ resulting in a slightly decreased *µ*_wt_, so the increased *n*_H_ is detrimental to electrical transport properties. The quality factor *β* at room temperature for all samples has been calculated to construct a relationship with the orientation factor *F* (Figure 7d). Both two methods could enhance the orientation factor and further optimize *β*. Evidently, the HF method presents a higher level of *β*, which is more beneficial to thermoelectric properties.

**Figure 8:**
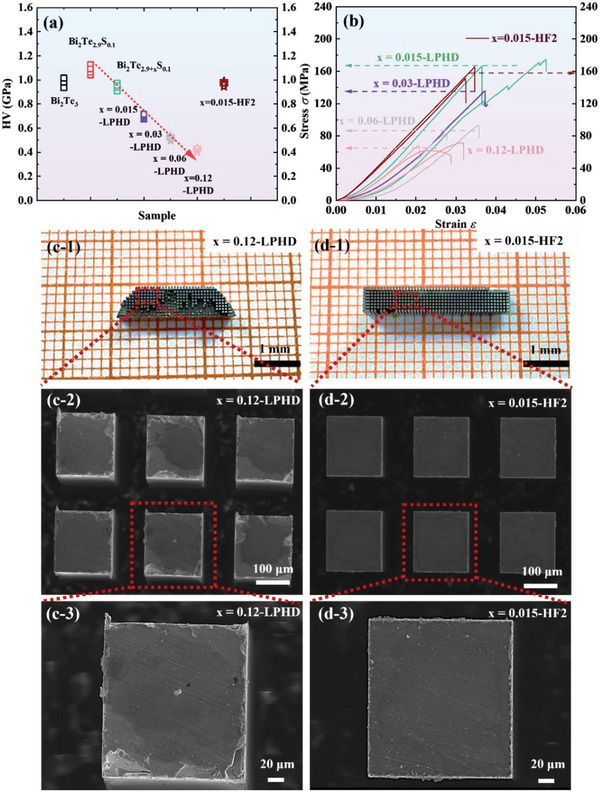
The a) Vickers hardness and b) compressive strength of LPHD and HF2 samples. c, d) The practical images and magnified SEM figures of the *x* = 0.12‐LPHD and *x* = 0.015‐HF2 samples after precision machining, respectively.

The Vickers’ hardness and compressive strength for all samples have been characterized for the potential application of micro‐refrigeration (**Figure** **8**a,b). Apparently, with the increase of Te content, the Vickers hardness of Bi_2_Te_2.9+_ *_x_* S_0.1_ samples shows a dramatic deterioration from 1.1 GPa to 0.4 GPa. Many factors, such as the low melting point eutectic phase, the porous structure, the grain growth, and the released strain, are related to the reduced rigidity of bulk samples. The *x* = 0.015‐HF2 sample has a relatively good Vickers hardness of 1.0 GPa which is comparable to that of *x* = 0.015‐ LPHD sample, despite the larger grain size. The balance between the recrystallization and work hardening, as well as the unchanged Te content, gives rise to comparable hardness. Likewise, the compressive strength results of LPHD samples also present a decreased tendency similar to the trend of hardness. Although some materials were squeezed out of the graphite die for *x* = 0.12‐LPHD sample, the residual eutectic phases are still distributed along the grain boundaries (Figure S5, Supporting Information). The weak grain boundaries induced by eutectic phases might be the main reason for the deteriorative mechanical properties. Expectedly, the compressive strength of *x* = 0.015‐HF2 sample exhibits 160 MPa that is also similar to the *x* = 0.015‐LPHD sample.

We further machined the samples finely, namely *x* = 0.12‐LPHD and *x* = 0.015‐HF2, to simulate the practical fabrication process of microdevices. The 97% production yield for successfully cutting thermoelectric legs of *x* = 0.015‐HF2 sample is remarkably higher than that of *x* = 0.12‐LPHD sample (Figure 8c,d). SEM images show the surface morphology of both *x* = 0.12–LPHD and *x* = 0.015‐HF2. It is distinct that mechanical damages of the surface quality and structure for *x* = 0.12‐LPHD sample have been observed after the machining process. These defects would bring risks in fabricating barrier coating and solder for assembling micro cooling devices. The poor quality of the surface structure would increase the contact resistance and therefore obviously reduce the device's performance. In contrast, the HF sample presents extraordinary processability without obvious damage on the leg surface. The next challenging step is about fabricating micro‐devices using high‐ZT materials prepared by the HF method, which is beyond the scope of our present investigation purpose. Consequently, compared to the liquid phase hot deformation, the hot forging method is a more feasible approach to yield n‐type bismuth telluride materials with high thermoelectric performance and robust mechanical properties for commercial micro‐refrigeration applications.

## 4 Conclusions

Liquid phase hot deformation and hot forging are effective strategies to tackle the low preferred orientation of n‐type Bi_2_Te_3_ materials. In this work, we, for the first time, systematically elucidated the critical differences in thermoelectric and mechanical properties between samples fabricated by these two methods. The hot forging method can effectively construct grain‐preferred orientation and concurrently stabilize the optimized composition, leading to the increased thermoelectric quality factor. Additionally, high dense dislocations in hot‐forging samples result in robust mechanical performance and good machinability in precision manufacturing. Therefore, this work is to prove the superiority of the HF method, for both thermoelectric and mechanical properties, which provides a feasible way to further optimize the thermoelectric performance of n‐type Bi_2_Te_3_ materials and also makes valuable contributions to commercial micro‐refrigeration applications.

## Conflict of Interest

The authors declare no conflict of interest.

## Author Contributions

Y.‐K. and Y. J. contributed equally to this work. Y.‐K. Z designed this work. Y.‐K. Z., Y. J., and X. D. prepared the thermoelectric materials and measured the transport properties. J. Z. and F. G provided the first principle calculations. Y.‐K. Z. and M. L analyzed the Rietveld refinement. Y.‐K. Z., F. L., and Y. S. measured the mechanical properties. All authors discussed the results and contributed to the data analyses. Y.‐K. Z., Z. L., and J. S. wrote and edited the manuscript. W. C., Z. L., and J. S. supervised the whole project.

## Supporting information

Supporting Information

## Data Availability

All data necessary to understand and assess this manuscript are shown in the main text and the Supporting Information.
