# Supplementary material for: Design of N‐Type Textured Bi2Te3 with Robust Mechanical Properties for Thermoelectric Micro‐Refrigeration Application
Source: Adv Sci (Weinh). 2022 Dec 29;10(6):2206395. doi: 10.1002/advs.202206395 (PMC9951298; doi:10.1002/advs.202206395)
Supplement: Supplementary file 1 — Supporting Information [file ADVS-10-2206395-s001.pdf]

ADVANCED SCIENCE
Open Access

Supporting Information

for *Adv. Sci.*, DOI 10.1002/advs.202206395

Design of N-Type Textured Bi_2Te_3 with Robust Mechanical Properties for Thermoelectric Micro-Refrigeration Application

Yu-Ke Zhu, Yifan Jin, Jianbo Zhu, Xingyan Dong, Ming Liu, Yuxin Sun, Muchun Guo, Fushan Li, Fengkai Guo, Qian Zhang, Zihang Liu, Wei Cai and Jiehe Sui**

Supporting information

Design of N-Type Textured Bi_2Te_3 with Robust Mechanical Properties for Thermoelectric Micro-Refrigeration Application

Yu-Ke Zhu[1#], Yifan Jin[1#], Jianbo Zhu[1], Xingyan Dong[1], Ming Liu[1], Yuxin Sun[1], Muchun Guo[1], Fushan Li[1], Fengkai Guo[1], Qian Zhang[2], Zihang Liu[1], Wei Cai[1], Jiehe Sui[1*]*

1. State Key Laboratory of Advanced Welding and Joining, Harbin Institute of Technology, Harbin 150001, China.

2. School of Materials Science and Engineering, Institute of Materials Genome & Big Data, Harbin Institute of Technology, Shenzhen 518055, China

Corresponding author: To whom correspondence should be addressed. E-mail: *zihangliu@hit.edu.cn; suijiehe@hit.edu.cn*

The orientation factor is calculated by the following equations:

$$F = \frac{P - P_0}{1 - P_0} \quad \text{(S1)}$$

$$P_0 = \frac{I_0(00L)}{\sum I_0(hkl)} \quad \text{(S2)}$$

$$P = \frac{I(00L)}{\sum I(hkl)} \quad \text{(S3)}$$

where P and P_0 are ratio of the integrated intensities of all (00*l*)-planes to the intensities of all (*hkl*) planes for measured sample and the randomly oriented sample, respectively. The calculated F value distributes in the range of 0~1, in which 0 means totally isotropic.

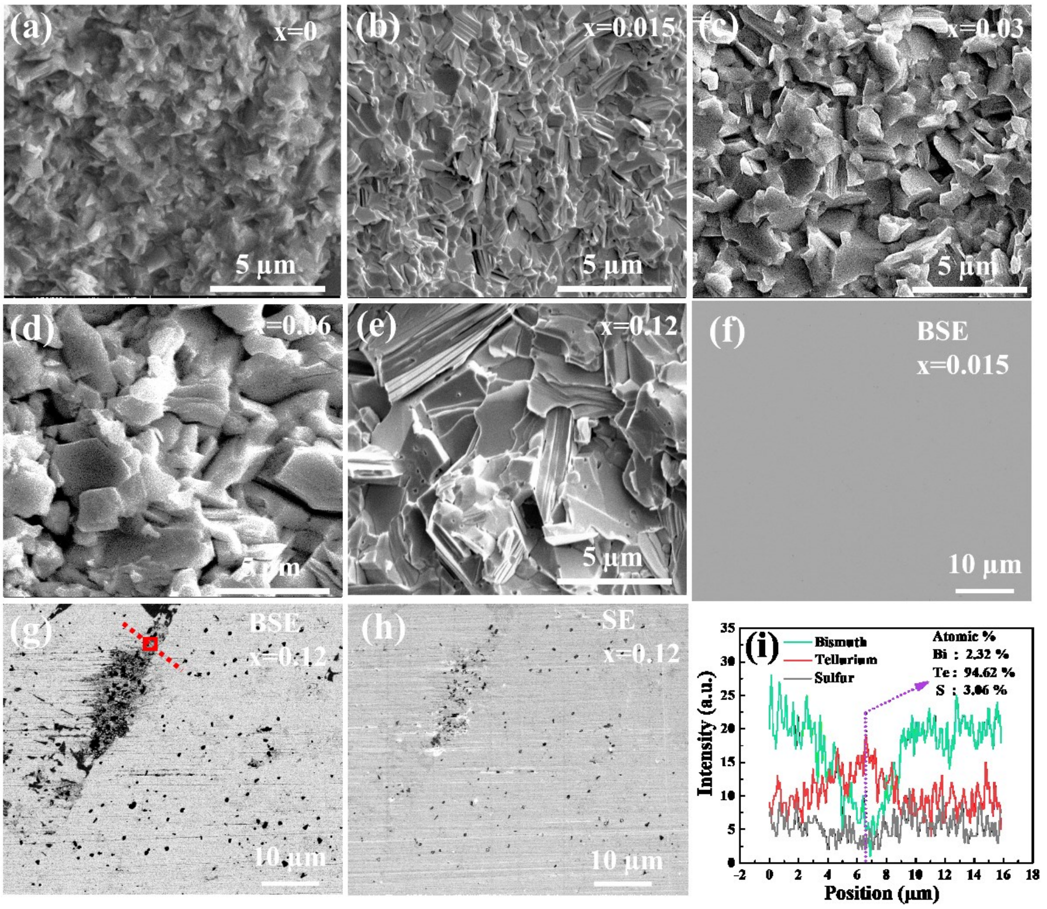

Figure S1. The a-e) SEM images of bulk $Bi_2Te_{2.9+x}S_{0.1}$-LPHD (x = 0, 0.015, 0.03, 0.06, and 0.12) samples, and f) the BSE images of the samples x = 0.015-LPHD and x = 0.12-LPHD. The h) secondary electron image of x = 0.12-LPHD sample and the i) EDS line and point scanning located on the red dash line in g).

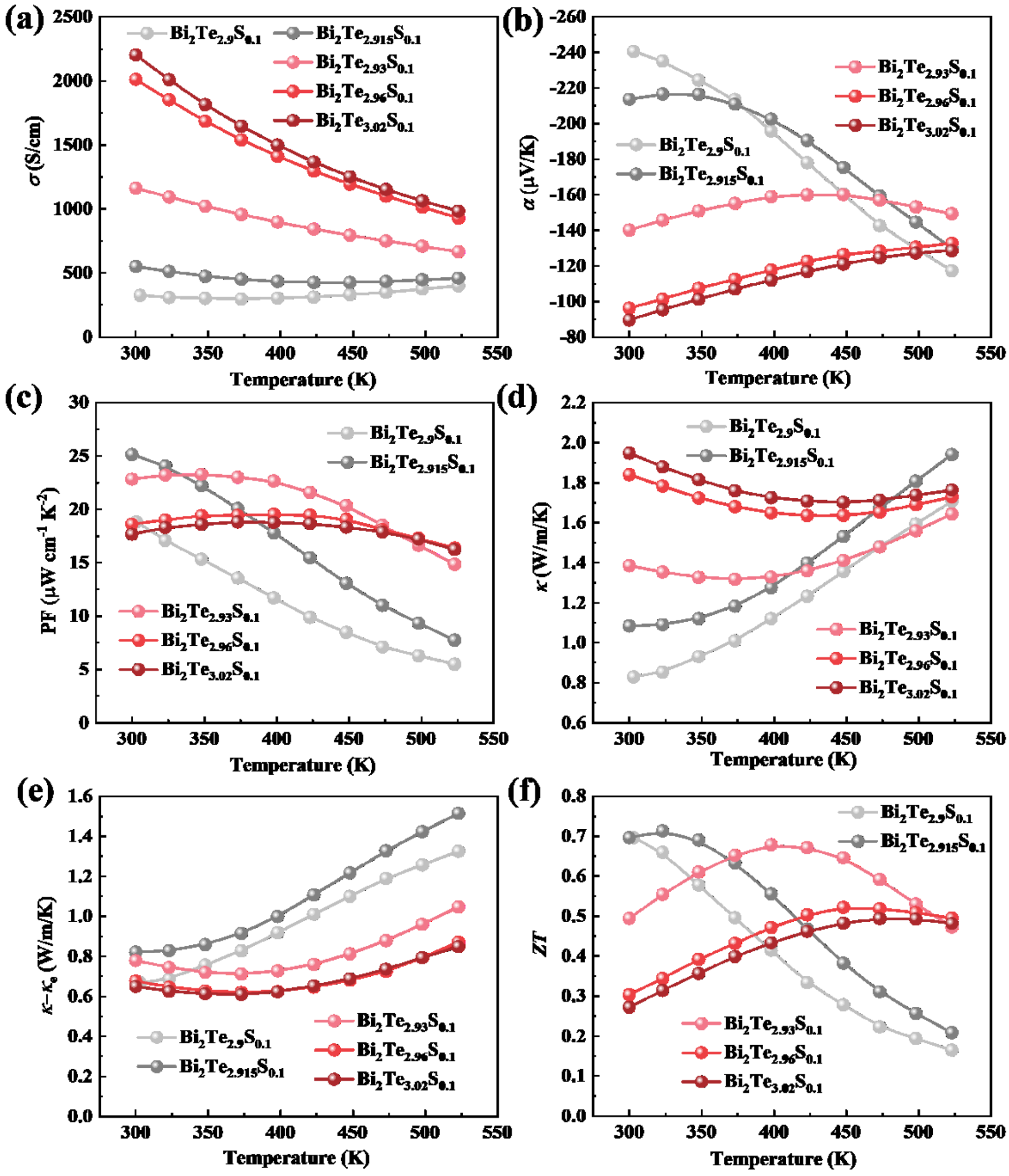

Figure S2. Temperature dependence of a) electrical conductivity σ, b) Seebeck coefficient α, c) power factor *PF*, d) total thermal conductivity κ, e) thermal conductivity of κ - κ_e and f) figure of merit *ZT* for $Bi_2Te_{2.9+x}S_{0.1}$-LPHD (x = 0, 0.015, 0.03, 0.06, and 0.12) samples.

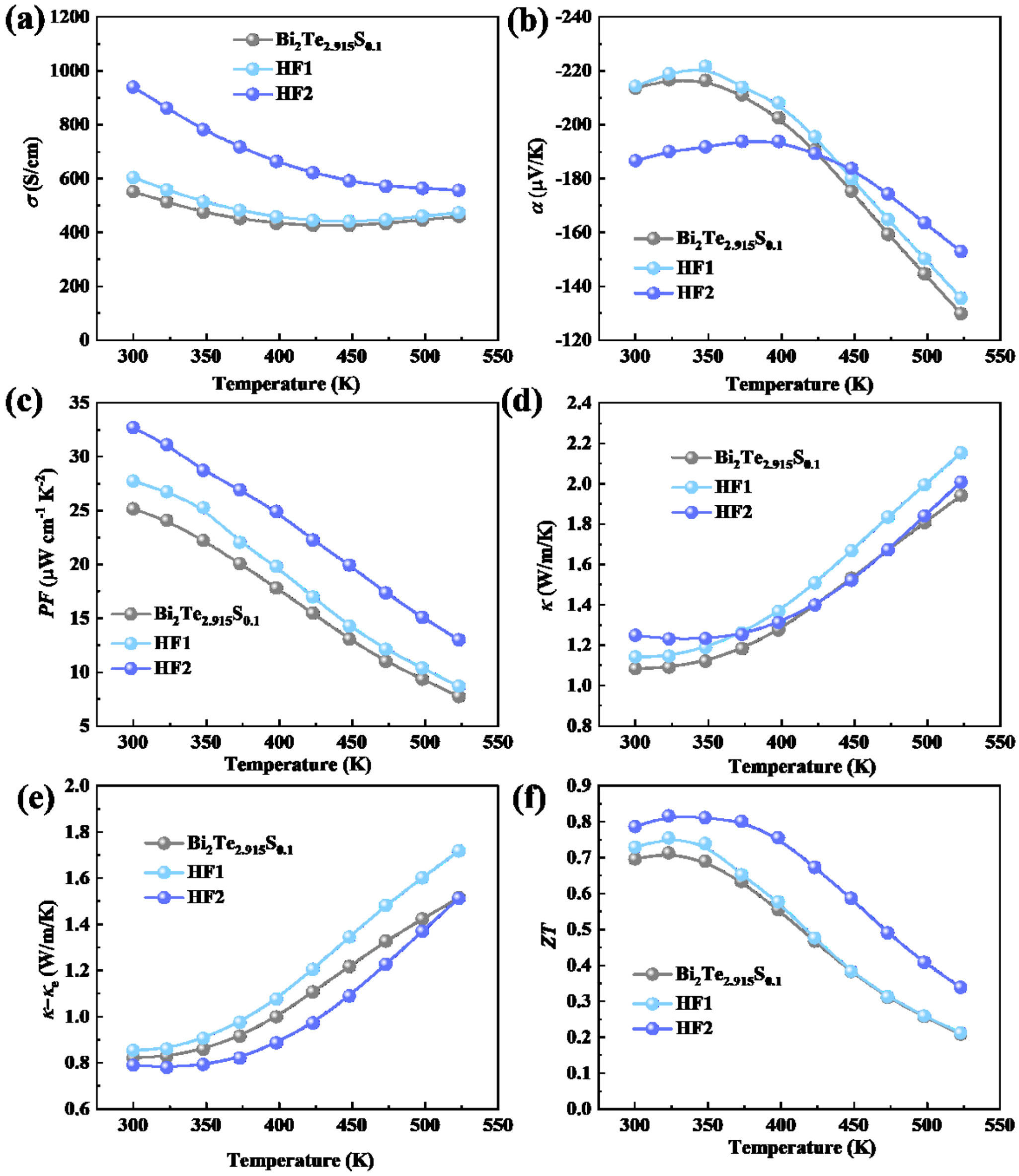

Figure S3. a) Temperature dependence of a) electrical conductivity σ, b) Seebeck coefficient α, c) power factor PF, d) total thermal conductivity κ, 5) thermal conductivity of κ - κ_e and 6) figure of merit ZT for x = 0.015-LPHD, x = 0.015-HF1 and x = 0.015-HF2 samples.

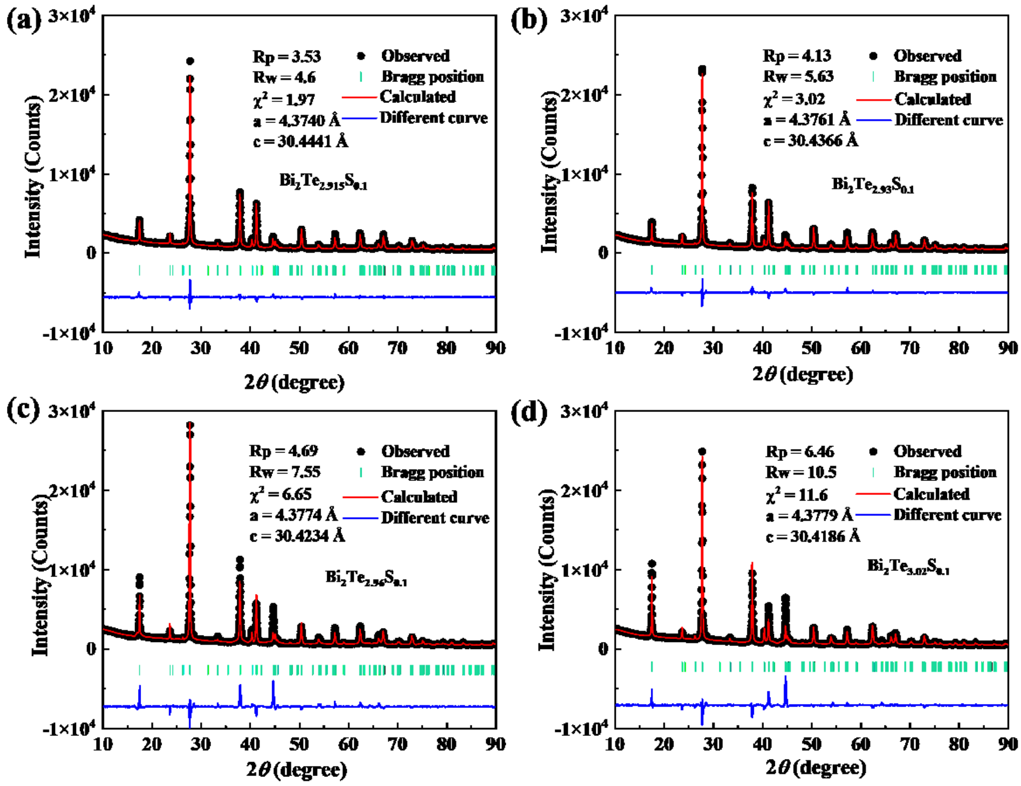

Figure S4. The Rietveld refinement results of $Bi_2Te_{2.9+x}S_{0.1}$-LPHD (x = 0.015, 0.03, 0.06, and 0.12) samples based on single phase.

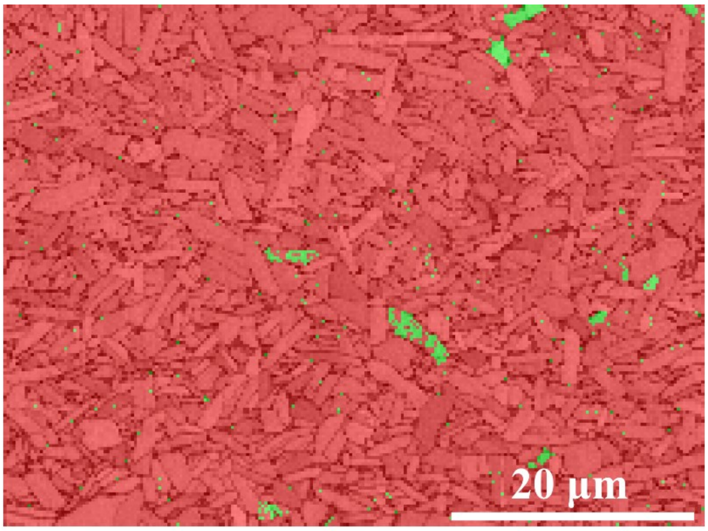

Figure S5. The detected phases distribution image of x = 0.12-LPHD sample corresponding to the Figure 3a. (Red for Bi_2Te_3, and green for Te)
